# Transcranial Electric Stimulation for Precision Medicine: A Spatiomechanistic Framework

**DOI:** 10.3389/fnhum.2017.00159

**Published:** 2017-04-13

**Authors:** Fatemeh Yavari, Michael A. Nitsche, Hamed Ekhtiari

**Affiliations:** ^1^Department of Psychology and Neuroscience, Leibniz Research Centre for Working Environment and Human FactorsDortmund, Germany; ^2^Department of Neurology, University Medical Hospital BergmannsheilBochum, Germany; ^3^Neurocognitive Laboratory, Iranian National Center for Addiction Studies (INCAS), Tehran University of Medical SciencesTehran, Iran; ^4^Translational Neuroscience Program, Institute for Cognitive Science Studies (ICSS)Tehran, Iran; ^5^Neuroimaging and Analysis Group, Research Center for Molecular and Cellular Imaging (RCMCI), Tehran University of Medical SciencesTehran, Iran

**Keywords:** transcranial electrical stimulation (tES), transcranial direct current stimulation (tDCS), application, protocol, montage, precision medicine, individualized, spatiomechanistic

## Abstract

During recent years, non-invasive brain stimulation, including transcranial electrical stimulation (tES) in general, and transcranial direct current stimulation (tDCS) in particular, have created new hopes for treatment of neurological and psychiatric diseases. Despite promising primary results in some brain disorders, a more widespread application of tES is hindered by the unsolved question of determining optimum stimulation protocols to receive meaningful therapeutic effects. tES has a large parameter space including various montages and stimulation parameters. Moreover, inter- and intra-individual differences in responding to stimulation protocols have to be taken into account. These factors contribute to the complexity of selecting potentially effective protocols for each disorder, different clusters of each disorder, and even each single patient. Expanding knowledge in different dimensions of basic and clinical neuroscience could help researchers and clinicians to select potentially effective protocols based on tES modulatory mechanisms for future clinical studies. In this article, we propose a heuristic spatiomechanistic framework which contains nine levels to address tES effects on brain functions. Three levels refer to the spatial resolution (local, small-scale networks and large-scale networks) and three levels of tES modulatory effects based on its mechanisms of action (neurochemical, neuroelectrical and oscillatory modulations). At the group level, this framework could be helpful to enable an informed and systematic exploration of various possible protocols for targeting a brain disorder or its neuroscience-based clusters. Considering recent advances in exploration of neurodiversity at the individual level with different brain mapping technologies, the proposed framework might also be used in combination with personal data to design individualized protocols for tES in the context of precision medicine in the future.

## Introduction

**Figure 1:**
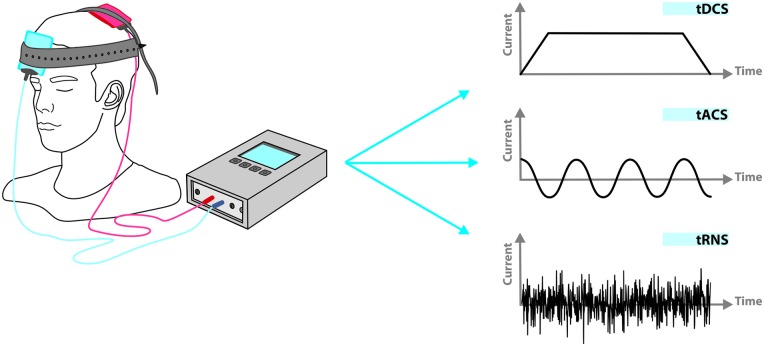
**Applied current in transcranial electrical stimulation (tES) can be direct (transcranial direct current stimulation, tDCS), alternating (transcranial alternating current stimulation, tACS), or random (transcranial random noise stimulation, tRNS).** Beyond current shape, other stimulation parameters such as duration, frequency and phase in relation to spontaneous neuronal activity can be adjusted independently.

Transcranial electrical stimulation (tES), as a non-invasive brain stimulation technique, consists of delivering weak electrical currents (~1–2 mA) to the head for several minutes (~5–30 min) via scalp electrodes. The applied currents can be direct (transcranial Direct Current Stimulation, tDCS), alternating (transcranial Alternating Current Stimulation, tACS), or random noise (transcranial Random Noise Stimulation, tRNS; Figure 1). tES in general, and tDCS in particular, have gained serious interest in recent years and created new hopes in various clinical applications. Preliminary promising results, obtained in different neurological and psychiatric disorders such as depression (Nitsche et al., 2009), post-stroke motor deficits (Kang et al., 2016), post-stroke aphasia (Baker et al., 2010), and pain (Lima and Fregni, 2008), suggest tES as a feasible therapeutic modality.

Despite tES appealing characteristics such as being affordable and easy-to-operate, myriad adjustable parameters necessitates further studies for identification of the most efficient protocols for each disorder, and even each individual before extending to routine clinical employment of tES. These parameters contain current type, amplitude, polarity (for DC current), phase (for AC current), electrode size, shape, number, montage and also duration, number and interval of stimulation sessions (Brunoni et al., 2012). Electrode montages in the published studies, *per se*, have been categorized into four groups according to their physical characteristics (Nasseri et al., 2015): (1) unilateral montages which target only one hemisphere; (2) bilateral montages which target both hemispheres; (3) midline montages which target region(s) under the midline; and (4) dual channel montages which employ two pairs of electrodes connected to two independent electrical circuits. This huge puzzle of parameters and their physiological and functional impact have been explored in a large body of basic and clinical studies on tES (Medeiros et al., 2012; Fregni et al., 2015; Nitsche et al., 2015).

**Figure 2:**
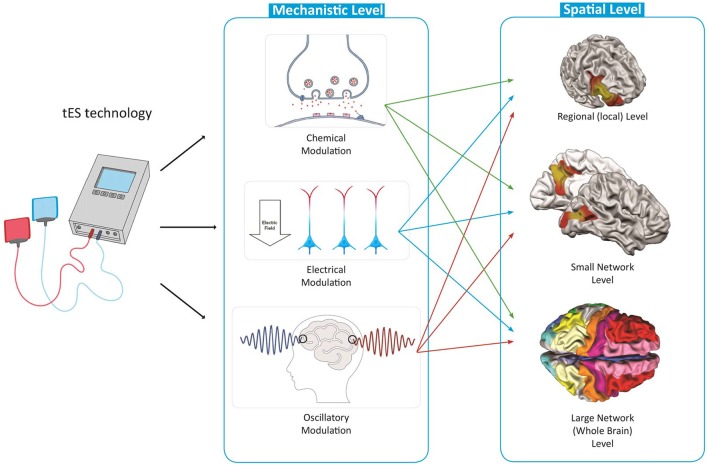
**Nine-level spatiomechanistic framework for systematic exploration of tES protocols**.

The large variety of the possible stimulation protocols limits the identification of the full clinical potential of tES and its implementation into everyday clinical practice. There is lack of a systematic way to narrow down possible protocols to potentially more efficient ones for each brain disorder based on neuroscientific evidence, and to build the foundation for large scale trials. In this article, inspired from the expanding neuroscience knowledge, we present a spatiomechanistic multilevel framework, which can be helpful as a guidance to explore various possible protocols and to make an informed selection between these for a target brain disorder (shown schematically in Figure 2). In this framework, we describe tES mechanisms of action based on three distinct, yet not independent, mechanistic levels: (1) neurochemical; (2) neuroelectrical; and (3) oscillatory. Each of these three mechanistic modulations are investigated for three spatial levels of the brain: (1) local (one brain area of interest); (2) small-scale networks (two connected brain regions); and (3) large-scale networks (whole brain level). For a given disorder, depending on its pathology, i.e., its neurophysiological alterations and spatial location and extension of these alterations, it is possible to describe and/or define neuroscience-informed stimulation protocols based on this spatiomechanistic framework. Beyond defining adapted protocols, this framework could also help to identify the gaps in the disease-related neuroscience knowledge relevant for informing a protocol in one of the nine levels.

We chose three spatial levels, namely local, small networks and large networks. Traditionally, insights into brain function have been obtained from studying individual brain regions. It was assumed that each brain area is responsible for a specialized function and different regions act relatively independent from each other. Advancement in data acquisition and analysis techniques has created increasing attention towards small-scale and large-scale brain networks in neuroscience studies during the past decade. Two anatomically/functionally connected regions form a small network in the brain (local networks, between two seeds). Distributed brain areas interact with each other and form large-scale networks (whole brain networks, between more than two regions). It has been suggested that complex brain functions emerge from these interactions (Shafi et al., 2012). Even psychiatric and neurological diseases have been suggested to be disorders of brain networks (Shafi et al., 2012; Fox et al., 2014). In some diseases a large network, consisting of several interacting and overlapping dynamic subnetworks, is mainly engaged. Malfunctioning of each subnetwork is appointed to a clinically separable aspect of that disease. An example is the tinnitus network with its subnetworks characterizing distress, sound features, lateralization, etc. (De Ridder and Vanneste, 2012).

In the following sections, we first review some neuroscientific evidence for tES effects at these nine levels. Then, we will explain how this framework might help to come to an informed definition of protocols suited for treatment of some brain disorders and their subtypes and how it might prospectively encourage designing individually tailored protocols in combination with individual brain mapping data.

## Mechanistic Levels of tES Effects

At each of the previously-mentioned spatial levels (local, small-scale networks and large-scale networks), the physiological response of the brain to tES can be explained based on its “neurochemical” or “neuroelectrical” consequences, or its effects on “brain oscillations or waves”. In the following, we go forward step by step by explaining each of the nine levels in the proposed spatiomechanistic framework and reviewing some relevant evidence in the basic and clinical neuroscience fields.

### tES and its Neurochemical Impacts

There is a micro-macro association between neurochemicals and various neural processes such as cortical plasticity. Different cognitive functions such as emotion, memory and even consciousness might be mediated by the complex interactions of many neurotransmitters. Various psychiatric disorders and neurodegenerative diseases have some roots in the dysfunction of neurotransmitter systems. Advancement of knowledge about brain neurochemistry may yield to better identification of the molecular basis of disorders and disease-specific biomarkers.

tES can affect brain neurochemistry; i.e., it can modulate molecular, cellular and biochemical aspects of the nervous system and mechanisms of molecular signaling and communication. Thereby, it influences the function of neurons and neural processing. tDCS modifies the synaptic microenvironment and regulates different neurotransmitters by modulating glutamatergic and gamma-Aminobutyric acid (GABA)-ergic activity (Liebetanz et al., 2002; Nitsche et al., 2003a, 2004a,b,c, 2012). Its long-lasting after-effects have been attributed to potentiation of synaptic glutamatergic receptors (Nitsche et al., 2003a, 2005), and are influenced by GABAergic neurotransmission via interneurons (Nitsche et al., 2004c; Stagg et al., 2009), and brain-derived neurotrophic factor (BDNF) secretion (Fritsch et al., 2010; Medeiros et al., 2012). An increase in BDNF (an important biomarker in synaptogenesis and neuroplasticity (Brunoni et al., 2012) secretion has been observed after tDCS and suggested to be a key mediator for long-lasting synaptic potentiation (LTP) induced by tDCS (Fritsch et al., 2010). It has also been shown that application of anodal direct current to the surface of the rat cortex increases early gene expression (Islam et al., 1995). Physiological mechanisms underlying the observed effects of tACS and tRNS remain active areas of research and might be slightly different, as, for instance, it has been shown that aftereffects of tRNS are not N-methyl-D-aspartate-receptor dependent (Chaieb et al., 2015).

These neurochemical alterations might happen in the regions underneath the stimulation electrodes, a distant area, or within widespread brain regions, as explained in the following sections.

#### Local Neurochemical Modulations by tES

Neurochemical changes induced by tES might happen just beneath the stimulation electrode and not in distant regions. Some examples are observations in studies which have examined the spatial extent of changes of brain metabolites using proton magnetic resonance spectroscopy (^1^H MRS), e.g., increased myoinositol concentration only underneath the anodal electrode placed over the right M1 (Rango et al., 2008), localized increase in the concentration of combined glutamate and glutamine within the right parietal cortex under the stimulating electrode (Clark et al., 2011; Hunter et al., 2015), and polarity-specific and localized reduction of the concentration of GABA (and not other key metabolites like Glutamate, Glutamine and N-acetylaspartate) by anodal stimulation of the left motor cortex (Kim et al., 2014).

#### Neurochemical Modulations of Small Brain Networks by tES

Other than its direct local effects on neurochemistry, tES can have a direct and/or indirect modulatory effect on the neurochemistry of remote areas. tDCS over the frontal cortex in the rat, for example, changed extracellular dopamine, but not serotonin, level in the striatum in a polarity dependent manner (cathodal, but not anodal; Tanaka et al., 2013) and it is speculated to cause similar effects in humans as well. In a study by Fregni et al. (2008), tDCS (anode over right and cathode over left dorsolateral prefrontal cortex (PFC)) reduced craving level of participants and fixation of food-related pictures. One speculative explanation for these observations is the stimulation of mesolimbic dopaminergic projections to the striatum and induction of dopamine release in the caudate nucleus. This might mimic reward and thereby eliminate the need for food intake (Fregni et al., 2008). Regulation of dopamine release in the striatum by transcranial stimulation of the cerebral cortex has been previously shown for repetitive transcranial magnetic stimulation (rTMS) and is suggested to be mediated through glutamatergic corticostriatal efferents. Strafella et al. (2003) used [^11^C]raclopride and positron emission tomography (PET) to measure changes of extracellular dopamine concentration in the putamen following rTMS of the motor cortex. They showed that rTMS of the left primary motor cortex leads to reduced [^11^C]raclopride binding potential in the left putamen which indicates focal dopamine release in this area (Strafella et al., 2003).

#### Neurochemical Modulations by tES at Whole Brain Level

Neurochemical and neurobiological findings suggest that tES can induce physiological alterations in extensive brain areas. For instance, application of tDCS (anode over the left motor and cathode over contralateral supraorbital cortices) resulted in a significant decrease in glutamate and glutamine within the anterior cingulate, a trend towards decreased glutamate and glutamine in the thalamus, and a trend towards increased GABA in the anterior insula (Foerster et al., 2015).

Several interleaved PET-tDCS studies have shown that motor cortex neuromodulation generates neurochemical regulations in broad regions of the brain (DosSantos et al., 2012, 2014; Yoon et al., 2014). For instance, in a study by DosSantos et al. (2014), PET scans acquired during anodal/cathodal modulation of right M1/contralateral supraorbital region (a montage which has been shown to produce analgesia effects) revealed changes in endogenous μ-opioid receptor-mediated neurotransmission within several regions including the periaqueductal gray matter, precuneus and left PFC. These changes have been attributed to the activation of the analgesic μ-opioid process (DosSantos et al., 2014).

In a phase II double-blind trial on subjects with chronic hepatitis C infection, five consecutive days of active tDCS (anode over the left primary motor cortex and cathode over the supraorbital right region) enhanced BDNF serum levels. This suggests that tDCS might promote neuroplastic changes in pain pathways including modulation of pain-regulating neurotransmitter release. BDNF is widely distributed in the central nervous system, has been suggested to be a possible neuroplasticity marker, and could act as a molecular marker of global neuronal activity. Therefore, tDCS, with the ability of regulating BDNF and other neurotransmitters in the plasma, could be considered as a modulator of global neural activity (Brietzke et al., 2015).

In another study, looking for beneficial consequences of tDCS on the 1-methyl-4-phenyl-1,2,3,6-tetrahydropyridine-induced mouse model of Parkinson’s disease, Lu et al. (2015) positioned the anodal stimulation electrode over the left frontal cortex and the cathodal electrode over the area between the shoulders. They observed that tDCS compensated for abnormal changes caused by 1-methyl-4-phenyl-1,2,3,6-tetrahydropyridine for the level of dopamine, enzymatic tyrosine hydroxylase, nonenzymatic malonaldehyde, enzymatic superoxide dismutase and glutathione peroxidase within the mouse brain. Accordingly, the authors suggested tDCS as a potential therapeutic modality for Parkinson’s disease (Lu et al., 2015).

### tES and its Neuroelectrical Impacts

Knowledge about the electrical excitability of the cerebral cortex is long-standing (Fritsch and Hitzig, 1870). Since neurons are electrically charged structures, extracellular electric fields affect their excitability. It is assumed that an electric field can change the permeability of biological membranes for different ions by affecting different neuronal membrane channels, such as sodium and calcium, and therefore alter the electrical conductance of the membrane. Depolarization/hyperpolarization of biological membranes and therefore increase/decrease of cortical neuronal excitability and spontaneous firing rates by anodal/cathodal stimulation is a well-accepted concept for the impact of transcranial direct current application on cerebral tissue (Nitsche and Paulus, 2000, 2001; Nitsche et al., 2003b). Obviously neurochemical and neuroelectrical consequences of tES are interrelated. Neuroelectrical modulations might take place in a specific brain region, a small network, or within widespread brain areas.

#### Local Neuroelectrical Modulations by tES

The primary effect of tDCS can be explained based on the non-invasive polarization of specific brain regions. The prolonged effects of the polarizing currents on the electrical activity of the rat cerebral cortex were demonstrated more than a half-century ago. Anodal stimulation increased neuronal firing, while cathodal stimulation resulted in reversed effects (Bindman et al., 1964). LTP- and Long-term Depression (LTD)-like effects induced by tDCS are probably initiated by neuronal depolarization or hyperpolarization. Online and offline effects of tDCS have been attributed to modulation of membrane potential during stimulation, and synaptic modification, respectively (Stagg and Nitsche, 2011). tACS, in a frequency- and state-dependent manner, and tRNS are also able to modulate cortical excitability, presumably by similar primary effects as tDCS, i.e., alteration of the membrane polarization (Terney et al., 2008; Kanai et al., 2010; Moliadze et al., 2012), although respective stimulation protocols do not induce neuroplastic after-effects in each case.

In most tES studies, electrode montages have been selected based on its neuroelectrical effects underneath the electrodes; for instance, based on the decrease of neural activity of the lesioned hemisphere after stroke, in many studies employing tDCS for stroke recovery, the anode has been positioned directly over the lesioned cortex to increase its activity (Schlaug et al., 2008). Other examples are auditory hallucinations which have been suggested to be associated with hyperactivity of the auditory cortex. Accordingly, cathodal tDCS has been employed to decrease the electrical activity of this region (Brunelin et al., 2014).

#### Neuroelectrical Modulations of Small Brain Networks by tES

tES makes it possible to remotely modulate the activity of different cortical and subcortical areas. Modulation of the activity in deep brain regions used to be possible only through pharmacological interventions or implanted electrodes. Transcranial stimulation techniques including tES exploit the connections between cortical and deep regions of the brain to induce changes in the activity of these regions (Chib et al., 2013). Here, we point out some studies as examples of small-network-associated tES effects.

Concurrent fMRI-tDCS studies suggest network-based effects of tES. For instance, using tDCS, fMRI and dynamic causal modeling, it has been shown that application of anodal tDCS over the left inferior frontal cortex (a key region in speech) during performance of a picture naming task affects the frontal naming network and reduces the Blood-oxygen-level dependent (BOLD) signal in both inferior frontal sulcus, and left ventral premotor cortex. Results of dynamic causal modeling revealed different excitatory and inhibitory connections between the ventral premotor cortex and inferior frontal sulcus with anodal compared to sham stimulation. Interestingly, a linear positive correlation was revealed between reaction time of the naming and dynamic causal modeling-derived values for ventral premotor cortex to inferior frontal sulcus connection; i.e., participant-specific DC-induced performance changes were related to the strength of this link (Holland et al., 2016).

Small-scale-network-inspired tES montages have been employed in various addiction studies as well (Conti and Nakamura-Palacios (2014), for example, applied bilateral tDCS over the dorsolateral PFC of crack-cocaine dependents. The dorsolateral PFC and anterior cingulate cortex have a strong structural interconnection (Barbas and Pandya, 1989); therefore, the applied current over the dorsolateral PFC might affect the anterior cingulate cortex through highly conductive white matter tracts. A significant decrease of anterior cingulate cortex activity after bilateral tDCS (left cathodal/right anodal) over the dorsolateral PFC was observed in this study. This result suggests that tDCS over dorsolateral PFC can directly augment cognitive control and indirectly modulate drug-related cue processing through affecting the anterior cingulate cortex in crack-cocaine dependent subjects (Conti and Nakamura-Palacios, 2014). In another study, Boggio et al. (2008) employed two different bilateral dorsolateral PFC stimulation montages to increase/decrease the excitability of the left/right dorsolateral PFC and vice versa in a group of alcohol dependent individuals. Interestingly, both montages led to significant decrease in alcohol craving compared to sham. This observation can be explained based on a small scale network framework stating that both montages disturbed the balanced activation of right and left dorsolateral PFC which is relevant for craving states (Boggio et al., 2008).

Another example is disruption of inhibitory connections between the regions in two hemispheres via the corpus callosum (interhemispheric inhibition) after stroke. It is thought that in this condition the healthy hemisphere exerts too much inhibitory influence on the ipsilesional hemisphere. This unopposed inhibitory force might hinder the recovery process of the affected hemisphere (Loubinoux et al., 2003; Nair et al., 2007; Takeuchi and Izumi, 2012). Small-scale network-based interventional models trigger the idea of applying cathodal tDCS to the non-lesioned hemisphere and anodal tDCS to the lesioned hemisphere. This might counteract the pathological dysbalance via simultaneously reducing the inhibitory tone over the damaged area and upregulating its excitability. Findings support the superiority of this bihemispheric montage by generating greater and longer-lasting effects compared to merely modulation of the ipsilesional or contralesional hemisphere (for a review see Gomez Palacio Schjetnan et al., 2013).

#### Neuroelectrical Modulations by tES at Whole Brain Level

Various studies present evidence for neuroelectrical modulatory effects of tES within large brain networks. Integrated PET-tDCS and fMRI-tDCS experiments provide direct evidence for widespread consequences of tES. In an fMRI-tDCS study, for instance, chronic stroke patients learned a motor skill in the supine position while receiving bilateral M1 stimulation. They performed the same task 1 week later inside the MRI scanner to evaluate both, the amount of motor skill retention and continued learning. Participants also performed another untrained task inside the scanner to investigate generalization from the trained to the untrained motor task. tDCS enhanced online and continued motor skill learning and generalization of performance enhancement to the novel task. Looking for the neural substrates responsible for the observed continued motor skill learning, the authors identified an in-charge focused motor network mostly inside the damaged hemisphere consisting of M1, supplementary motor area, dorsal premotor cortex and the contralesional cerebellum. It seemed that tDCS was able to incline brain activation toward the normal pattern, i.e., more focused recruitment within the lesioned hemisphere instead of extensive bihemispheric employment (Lefebvre et al., 2015). Similarly, in a PET-tDCS study, *H_2_^15^O* PET of regional cerebral blood flow after anodal and cathodal stimulation (target electrode over left M1 and return electrode over the right frontopolar cortex) showed significantly modulated regional cerebral blood flow (local neuronal activity) in extensive cortical and subcortical areas including the left M1, right frontal pole, right primary sensorimotor cortex and posterior brain regions under both stimulation variants compared to sham (Lang et al., 2005).

In a study conducted on smokers, Meng et al. (2014) selected the bilateral frontal-parietal-temporal association area as the neural target and attentional bias as the cognitive function of interest and observed attenuated smoking behavior after tDCS. This result was explained based on a large-scale network concept, as application of cathodal stimulation to frontal-parietal-temporal cortices can affect areas such as insula, hippocampus and lateral PFC, which have a well-known role in addictive behaviors. Inhibiting the activity of the hippocampus and insula might suppress smoking-related contextual memories and thus the urge of the patients to use drugs (Bonson et al., 2002; Meng et al., 2014). Furthermore, inhibiting activity of the dorsolateral PFC might reduce drug cue-related attention (Meng et al., 2014).

For evaluating tES neuroelectrical aftereffects on large-scale networks, computational modeling approaches play a significant role. Computational forward models, which are used to delineate brain current flow and density distribution according to the individual anatomy and tissue properties, have attracted considerable attention in the tES domain. An expanding number of these modeling studies, based on simple spherical head models in the early studies and realistically shaped head models derived from MRI in more recent ones, have aimed to obtain the distribution of transcranially applied electrical current within the whole brain. These computational forward models have sometimes even challenged the traditional simplified assumption that the maximum stimulation effect happens “under” the electrodes. These models have great potential for defining hypotheses about current effects, but require physiological validation to make them useful for empirical experimentation.

Another important category of computational methods which have been employed in tES studies focuses on the analysis of connectivity within complex brain networks. Brain connectivity (pattern of anatomical, functional, or effective connectivity between distinct neural elements) is crucial to explain how neurons and neural networks process information. Electrophysiological and neuroimaging techniques such as resting state-fMRI have been used to acquire data for the analysis of interconnections linking various brain regions. These datasets (usually recorded before and after tES application) combined with computational connectivity analysis methods have been employed to reveal tES-induced alternations of the architecture and connectivity of human brain functional networks at the large scale level. In a related study, anodal/cathodal stimulation of M1/contralateral frontopolar cortex resulted in an alteration within some cortico-subcortical functional networks; i.e., it created a connectivity-driven modulation of functional coupling between stimulated M1 and thalamus, and between striatum and the main components of the default mode network. Attenuation of connectivity between default mode network elements has been speculated to be associated with the activation of motor task-related cortico-subcortical functional networks (Polanía et al., 2012b). A study by Chib et al. (2013) showed that anodal tDCS of the ventromedial PFC along with cathodal stimulation of the dorsolateral PFC (but not stimulation of only one of these areas) affects a large network containing ventromedial PFC, dorsolateral PFC, striatum and ventral midbrain, created significantly enhanced connectivity between the PFC and ventral midbrain and in turn increased subjective appraisals of facial attractiveness.

### tES and its Impact on Brain Oscillations

In recent years, numerous studies have demonstrated a close association between brain oscillations and cognitive functions (Uhlhaas et al., 2009). Likewise, abnormalities of neuronal synchronization and cognitive dysfunctions are closely correlated. Various disorders, including schizophrenia, epilepsy, autism, Alzheimer’s and Parkinson’s disease have been associated with abnormal temporal neural coordination (Bianchi et al., 2012).

tES provides the intriguing opportunity to modulate brain oscillations and thereby to influence cognitive processes. Even though tDCS works with direct electrical current, it has been shown to have the ability of modifying the power of different frequency bands of brain waves (Keeser et al., 2011; Jacobson et al., 2012). tACS is able to change the amplitude, frequency, or phase of electroencephalography (EEG) oscillations and modulate inter-areal neural synchronization. It can modulate brain oscillations in a frequency- specific manner and thereby influence cognitive processes (for a review see Herrmann et al., 2013; Woods et al., 2016). tRNS, which can be considered a specific type of tACS, was introduced in 2008 (Terney et al., 2008). It consists of application of randomly oscillating currents in a wide range of frequencies (e.g., between 0.1 Hz and 640 Hz). It has been suggested that tRNS modulates cortical excitability by interfering with ongoing neural oscillations in the cortex (Ho et al., 2013). Another possible mechanism for its observed effects is the induction of stochastic resonance in the brain by increasing the level of noise (Fertonani et al., 2011).

Alterations in brain rhythms by tES might happen locally, in a small brain network, or propagated within numerous areas.

#### Local Oscillatory Modulations by tES

The modulatory effects of tES on the brain rhythms has local components, such as a specific increase in theta and delta power within the cathodally polarized motor cortex (Ardolino et al., 2005), or a decrease in the beta and gamma power in the occipital cortex after cathodal tDCS application to this region (Antal et al., 2004). Electrophysiological evidence suggests that tACS, as a periodic external drive, can also modulate ongoing rhythmic brain activity and induce entrainment of brain oscillations in a frequency-specific manner. For instance, application of 10 Hz tACS to the parieto-occipital cortex increased alpha activity within this area (Helfrich et al., 2014).

There are some relevant modeling studies which simulated the response of a network of neurons to an external electrical field. These network/neuronal models improve our understanding of the underlying action mechanisms of tES, help us to interpret some observed phenomena in experiments, and to optimally individualize the stimulation parameters (for a review of some models, see Herrmann et al., 2013). For instance, simulation of the response of a network of pyramidal neurons and inhibitory interneurons to DC and AC fields demonstrated that the degree of entrainment of neural oscillations depends on the frequency of the applied field (Fröhlich and McCormick, 2010).

#### Oscillatory Modulations of Small Brain Networks by tES

The modulatory effects of tES on brain rhythms might lead to the synchronization of neural oscillations between two distal regions. Phase synchronization in different bands of brain waves (theta, alpha, beta and gamma) has been proposed as an important communication mechanism across different cortical regions. tACS has been successfully used to entrain oscillatory activity in the circumscribed cortical areas and exogenously boost the coupling between different cortical regions within a specific frequency band. In a study by Polanía et al. (2012a), in-phase and anti-phase 6 Hz tACS over the left prefrontal and parietal cortices, which is suggested to induce theta synchronization and desynchronization between these regions, had improving/deteriorating effects on the performance in a working memory task. This effect was interpreted as evidence for the causal relevance of theta phase-coupling between prefrontal and parietal areas for working memory performance in healthy humans (Polanía et al., 2012a). In another study, application of bihemispheric anti-phase tACS over occipital-parietal areas in the gamma frequency band (40 Hz) elevated interhemispheric coherence (phase synchronization) which in turn altered visual perception (Strüber et al., 2014).

#### Oscillatory Modulations by tES at Whole Brain Level

Modulation of brain rhythms by tES can have an effect on extensive regions of the brain. An example is the study by Ozen et al. (2010) who applied tES with a sinusoid waveform (0.8, 1.25 or 1.7 Hz) and performed extracellular and intracellular recordings from neocortical and hippocampal neurons in rats. Entrainment of neuronal activity by tES was observed in both cortical regions and distant hippocampal sites. Distant neurons might be affected directly by tES, or activated by polysynaptic pathways involving neurons in the neighborhood of the stimulating electrodes (Ozen et al., 2010). These results might be transferable to human research. It has been shown that anodal, but not cathodal, tDCS over the right posterior parietal cortex, with an extracephalic return electrode, has a modulatory effect not only on the parietal areas, but also on the noncontiguous synchronized frontal areas. It is noteworthy that the observed effects were limited to the alpha rhythm band, which was attributed to the relaxed state of participants (reduced information processing in the brain).

In a study by Polanía et al. (2011), EEG signals were recorded from 64 channels while subjects performed simple voluntary hand movements before and after the application of 10 min anodal tDCS over the left M1. Synchronization of regions involved in performance of the motor task (premotor, motor, and sensorimotor areas) was significantly increased via tDCS only in the task-related high-gamma (60–90 Hz) frequency band (Polanía et al., 2011).

Modeling approaches can be useful to interpret and predict EEG alternations induced by various stimulation configurations. In a modeling study, Merlet et al. (2013) simulated the effect of tACS over occipital regions on brain activity. They simulated the response of a population of neurons oscillating with alpha frequency (10 Hz) to transcranial sinusoidal stimulation with frequencies from 4 Hz to 16 Hz. Simulated EEG signals at 20 scalp electrodes showed significant increase of alpha power in the most left and right channels, more pronounced in the central and posterior channels, and only for tACS frequencies from 8 Hz to 12 Hz. The dependency of the results from the stimulation frequency has been explained based on the resonance of the neuronal “natural” frequency with the applied stimulation frequency. Beyond confirmation of the results of similar human studies, this model also predicted some changes in the previously not-recorded EEG channels, which were even more pronounced compared to the previously recorded occipital channels underneath the electrodes. This prediction could inform future experimental works. Such modeling approaches also create the possibility of exploring instantaneous effects of tACS on EEG activity which, because of the presence of the stimulation artifacts, is difficult to perform in an experimental set-up (Merlet et al., 2013).

Computational approaches for inferring brain connectivity and functional networks based on oscillatory activities reflected in EEG and magnetoencephalography (MEG) data fall into this category as well. Functional connectivity between regions can be estimated based on the coherence between recorded EEG signals from the two regions. A combined tDCS-EEG study by Notturno et al. (2014), for example, demonstrated that modulating the activity of a major cortical hub in the motor network (i.e., primary motor cortex) during a specific brain state (while subjects were performing a finger tapping task) can alter the functional architecture of the whole network. Specifically, it caused significant increase in beta and theta band coherence between activity of the stimulated M1 and sensorimotor cortices, and parietal and prefrontal cortical areas. Oscillations in the beta band have been linked to motor and sensorimotor functions and theta band waves are speculated to be involved in neural representations of hand kinematics (Notturno et al., 2014).

### Interaction between the Nine Levels of the Framework

As mentioned previously, the nine levels of the framework are interdependent, and not isolated from each other. In principle, no intervention can claim to exclusively exert influence on only one level, rather effects are often present across multiple levels. The main target of every intervention, which is defined based on the pathophysiology of the disorder, is in most cases restricted to one level (e.g., to modulate pathological oscillations, maladaptive plasticity, or a low level of dopamine in certain synapses), however, there are usually alterations at other levels as well, which can be secondary. Pharmacological interventions, for instance, are designed primarily based on their neurochemical effects, but neurochemical changes are accompanied by alterations of neuroelectrical and oscillatory properties of the nervous system as well. For example, the main symptoms of Attention Deficit Hyperactivity Disorder (ADHD) have been suggested to arise from decreased dopamine concentration primarily in the PFC (local, neurochemical abnormalities; Arnsten and Castellanos, 2011). Methylphenidate (Ritalin), the most common treatment for this disorder, is able to reduce dopamine re-uptake, thereby increasing the concentration of dopamine within the synaptic cleft and addressing associated symptoms of the disorder (Solanto, 2002). Although the primary and causal consequences of Ritalin are at a local neurochemical level, it also has larger-multi-level effects. Quantitative EEG analysis (Merkel et al., 2000; Song et al., 2005), and EEG and MEG data (Wienbruch et al., 2005; Korostenskaja et al., 2008) have demonstrated its ability for changing brain rhythms at local, small network and large network levels. Furthermore, methylphenidate can induce neurochemical changes in local, small networks, and large networks. Neurochemical changes in small networks have been observed in PET data showing that methylphenidate can induce changes of dopamine metabolism of the nigrostriatal pathway (Schabram et al., 2014). Neurochemical changes in large networks have been observed in PET data showing that methylphenidate can induce significant DA increases in striatum, amygdala and the medial orbitofrontal cortex (Volkow et al., 2013). EEG, TMS and fMRI studies have further demonstrated the ability of methylphenidate to modulate neuroelectrical properties at different spatial levels (Hoegl et al., 2011; Silberstein et al., 2016). Future studies are needed to elucidate these interactions before tES protocols that take these interactions into account can be designed.

**Figure 3:**
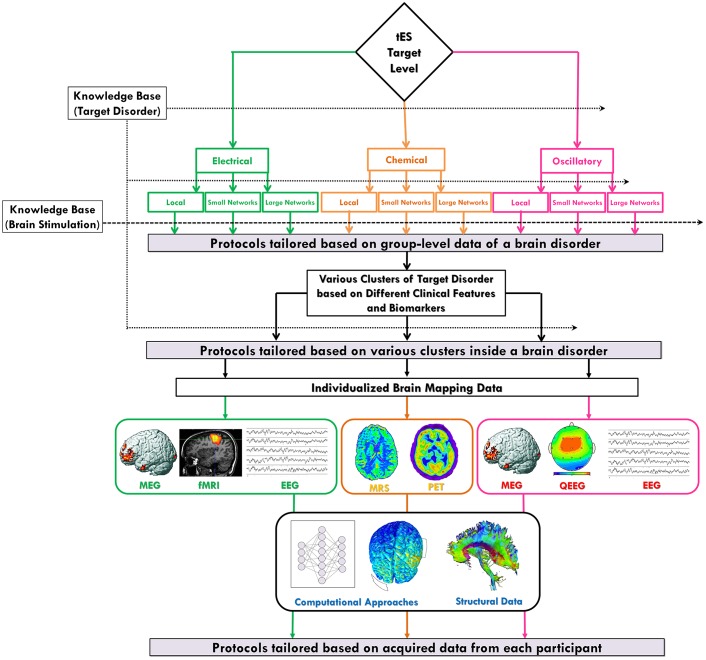
**Individualized protocol selection/definition based on the neuroscience-informed framework.** The proposed spatiomechanistic framework can guide tES users through individualized protocol selection/definition through three stages: (1) tailoring based on group-level data of a brain disorder: looking into the current knowledge base about the target disorder can provide some pieces of evidence to being narrowed down to one of the nine levels in the framework, as the most relevant one, before protocol selection/definition; (2) tailoring based on various clusters of a brain disorder: evidence might suggest existence of several subtypes of a particular disorder each requiring a different kind of tES protocol; and (3) tailoring based on individual-level data: neuroimaging or electrophysiological data obtained from each individual might provide valuable information for participant-specific protocol definition. Also, independent from the selected level of the framework, brain structural data and computational approaches can be helpful in this stage of tailoring process. Green, orange and pink colors are used to show pathways related to neuroelectrical, neurochemical and oscillatory levels, respectively.

Modulations which are produced by a neural intervention at different levels are an integrated phenomenon; however, in most of the existing studies, the question/concept of interest is focused on only one of the levels. Furthermore, current brain mapping techniques and analysis methods mostly generate data which are restricted to only a single level. Therefore, current knowledge bases (Figure 3) have a layered structure within the nine levels of the proposed spatiomechanistic framework. To assemble integrated data about changes in neuroelectrical, neurochemical and oscillatory properties of the human brain regions and networks is still a “work in progress” in neuroscience. Considering these limitations, the proposed framework is aimed to aid a structured protocol design/selection based on the existing multi-level body of evidence, and to move towards individualization by employing current brain mapping techniques.

## Towards Individualized tES Interventions

In one sense, medicine has always been personalized; because a decision about a specific treatment approach is usually made by integration of signs and symptoms, evidence, experience of the medical doctor and patient preference. On the other hand, interventions are approved based on the “groupwise” analyses of results of randomized clinical trials; i.e., most therapeutic interventions are designed for the “average patient” following a “one-size-fits-all” strategy (Ashley, 2015). The same intervention, however, does not have identical effects in all patients and consequently treatments can be very successful for some patients, but not for others. Some possible causes of this heterogeneity, especially for neurological and psychiatric disorders, are interindividual and even intra-individual biological differences, as well as state-dependent and non-linear effects of neuromodulatory interventions. Effects of tDCS, like other neuromodulatory brain stimulation interventions, show interindividual heterogeneity even when using identical stimulation parameters and applying them to healthy populations. Numerous neurodiversity-producing factors such as anatomy, genetics, age and organization of local inhibitory and excitatory circuits might contribute to this observation (Li et al., 2015). Intra-individual reliability of responses to tES has also been explored in different studies (Monte-Silva et al., 2010, 2013; Alonzo et al., 2012; Gálvez et al., 2013; Jamil et al., 2017), and might be affected by factors such as circadian, metabolic, hormonal cycles, or even methodological limitations such as variations in TMS coil position and orientation in same subject in different session (Ridding and Ziemann, 2010). With respect to this relationship, the large-scale parameter space in tES can provide an opportunity for designing individualized treatment protocols.

Precision or individualized medicine has gained increased importance in different clinical applications, especially in oncology. This approach, which often includes selecting optimal therapies based on the context of a patient’s genetic characteristics or other molecular analyses, tries to match specific treatments with the optimally suited patients and might relevantly alter the future of healthcare. Key contributing factors in the development of precision medicine include emerging biomedical technologies, powerful methods for characterizing patients, and computational approaches for analyzing large data sets. With the advancement in understanding the nature of various disorders, designing precisely tailored treatment approaches will gain increased importance.

Progression toward the era of precision oncology encourages personalized medicine respecting other diagnostic criteria and therapeutic strategies as well. Information employed in precision medicine often involves panomic (genomics, proteomics, metabolomics, transcriptomics and diverse cellular assays) data, but can also include other personal biomedical information across many layers, from molecular levels to behavior. These can incorporate clinical, behavioral, physiological and environmental parameters such as polymorphisms, anatomy, age, health history, lifestyle and diet. Tools employed in precision medicine can include molecular diagnostics, imaging, software/analytics, and methods for using large datasets. Many of these data types and tools can be relevant when thinking about the development of precision medicine in tES applications. Specifically, cutting edge and emerging brain mapping technologies, including state-of-the-art neuroimaging and electrophysiological devices, can provide valuable information about temporal, spatial, and other aspects of neural states, and might offer approaches towards the discovery of clinically valuable diagnostic, prognostic and therapy-outcome-predictive biomarkers. Therefore, in this section, we focus on the potential applications of brain mapping technologies for tES individualization.

The proposed framework, inspired from new advances in neuroscientific knowledge about tES action mechanisms, could offer a systematic strategy to explore the tES protocol space, make a more informed selection of protocols, and propose new ideas about designing participant-tailored protocols. Protocol individualization has the potential benefit of improving response and avoiding waste of time according to patient treatment with ineffective therapies. In this section, we describe how the proposed framework can provide a rationale to produce hypotheses about physiologically-based optimized/tailored stimulation protocols in three stages: (1) tailoring based on the group-level data of a brain disorder; (2) tailoring based on various clusters of patients with a brain disorder; and (3) tailoring based on individual-level data. In the previous sections, we have focused on the first two stages. In this section, we review them and introduce the third stage. The whole procedure is summarized in Figure 3 and explained in detail in the following paragraphs. The actual effectiveness of every suggested protocol by this framework undoubtedly needs to be verified in a new generation of evidence-based clinical trials before translating it from bench to the bedside in clinical settings.

### Tailoring Based on Group-Level Data of a Brain Disorder

This article aims to support researchers in different scenarios of clinical trial design. This includes proof of concept studies to evaluate the efficacy of tES for the treatment of a brain disorder, but also studies aimed to enhance stimulation outcomes compared to previous tES studies targeting the same disorder. In either case, one of the key steps in experimental design is to select an appropriate stimulation protocol. The proposed framework in this article recommends to look for answers to the two following main questions based on the available empirical evidence:

1. Is tES going to be used to affect brain neurochemistry, its neuroelectrical aspects, or its rhythms?
2. What is the spatial extent of the target that is intended for modulation? A specific region, a small network, or a large network in the brain?

To answer these questions, the knowledge base about the target disorder is essential. The following aspects might be especially relevant: “Is there any specific brain region involved in this disorder?”; “Is this region directly accessible for transcranial local stimulation or should it be accessed indirectly by modulating a cortical node within a network?”; “Does this disorder change some neurochemicals in the brain? If yes, how large is the spatial extension? Are these alterations limited to a specific region, involve a small network or even the whole brain?”; “Is there some reduction or increase of the activity in a region, small network or large scale network?”; “Does evidence demonstrate the presence of pathological neural oscillations? If so, do they occur within a brain region or a network?” To date, most clinical studies have addressed only one or two of these questions. However, to advance clinical translation of tES, future studies need to address most/all of these questions in a comprehensive manner using multiple approaches and analyses.

Gathering this information will result in identification of one of the nine levels of the framework as the “most relevant” one (which obviously will not be exclusive). The next step would be to identify a potentially effective protocol by examining the current knowledge base of brain stimulation and specifically tES studies with special attention to various employed protocols and underlying action mechanisms, and to identify a potentially effective protocol. In a recently published article, tDCS montages have been categorized in a framework of four groups. This framework can provide useful insights for montage selection at this stage (Nasseri et al., 2015). For instance, for targeting the brain at the local level, “unilateral monopolar” and “midline monopolar” categories might be the preferred classes of electrode montages. In what follows, we describe some examples of tailoring an intervention protocol based on group-level data at different levels of the proposed framework.

Stimulation of motor cortex using an implanted stimulation device has been shown to be a valuable analgesic intervention in patients with chronic neuropathic pain (Carroll et al., 2000). Its effects have been speculated to be caused by modulation of first and second order somatosensory areas and thalamic nuclei (Canavero and Bonicalzi, 2007). In accordance, single and multiple sessions of high-frequency (excitatory), but not inhibitory, rTMS over the precentral (motor) cortex has analgetic effects and generates relief of some types of chronic pain (Lefaucheur et al., 2001; Khedr et al., 2005; Lefaucheur, 2006). The underlying mechanisms have been attributed to increased activity of specific thalamic nuclei (via projections from the motor and premotor cortices), and consecutive activity alterations in the medial thalamus, anterior cingulate and upper brain stem (via a cascade of synaptic events; Khedr et al., 2005). These data can be associated with the “neuroelectric/large-scale networks” level in the spatiomechanistic framework. Following this concept, anodal tDCS over M1, with the return electrode placed over the contralateral supraorbital area, (a “bilateral bipolar-nonbalanced” montage Nasseri et al., 2015) can be suggested as a montage to be employed for pain reduction. Its proposed mechanism would be direct upregulation of cortical excitability, and/or indirect modulation of the pain-related structures such as thalamic and subthalamic nuclei, anterior cingulate, periaqueductal gray and spinal cord (Fregni et al., 2006b; Kuo et al., 2014). To select the stimulation target based on such a large scale network perspective is not a new idea; for example, internal globus pallidus, supplementary motor area, and premotor cortex, which have been selected as stimulation targets in dystonia, all pertain to the networks implicated in movement (Fox et al., 2014).

As another example, neuroimaging studies revealed pathologically reduced/increased activity of the left/right dorsolateral PFC in major depression (for a review see Kuo et al., 2014). These alterations are compatible with a neuroelectrical/local level intervention approach according to the spatiomechanistic framework. Anodal/cathodal tDCS can induce long-lasting enhancement/reduction of cortical excitability and activity (Nitsche and Paulus, 2000, 2001; Nitsche et al., 2003b; Monte-Silva et al., 2010, 2013). Therefore, it is possible to suggest montages to neuroelectrically modulate the relevant regions. Enhancement of excitability of the left dorsolateral PFC using anodal stimulation, with the cathode placed over the contralateral supraorbital region (a bilateral bipolar-non balanced montage, Nasseri et al., 2015), can improve depressive states (Fregni et al., 2006a). An even more promising montage might be bihemispheric stimulation (a bilateral bipolar-balanced montage) to simultaneously enhance excitability of the hypoactive left, and reduce the excitability of the hyperactive right dorsolateral PFC (Nitsche et al., 2009). Studying the interdependence of these spatial mechanisms may help to fine-tune the stimulation protocol within a precision medicine framework.

It has been suggested that the regional cortical excitation/inhibition balance, determined by the ratios of glutamate/GABA levels, plays a critical role in normal cognition (Krause et al., 2013). An alteration of this ratio, which has been speculated to be related to behavioral and cognitive deficits (Yizhar et al., 2011), has been demonstrated in some disorders such as autism, schizophrenia and ADHD (Rubenstein and Merzenich, 2003; Perlov et al., 2009). Particularly, increased glutamate level, and accordingly an altered excitation/inhibition ratio, has been observed in the frontal area of individuals with ADHD (for a review see Perlov et al., 2009). This concept is relevant to the neurochemical/local level in the proposed spatiomechanistic framework. tDCS is able to induce polarity-specific neurochemical changes in the cortex. Anodal tDCS causes locally reduced GABA activity, while cathodal stimulation reduces glutamatergic neurotransmission (Stagg et al., 2009). These concepts support the idea that the application of cathodal tDCS over frontal regions might restore the pathologically altered excitation/inhibition balance and have some beneficial effects for this patient population (Bandeira et al., 2016).

The following sections contain some hypothetical tES protocols based on the neurochemical, neuroelectrical, and oscillatory levels of the proposed spatiomechanistic framework, respectively.

#### Hypothetical tES Protocols Based on the Neurochemical Level of the Spatiomechanistic Framework

The proposed framework in this article can be helpful for suggesting tES protocols based on group-level data related to a brain disorder. Existing neuroscience knowledge about a target disorder can be utilized for getting narrowed down to one of the nine levels of the framework as the most relevant one. Then, depending on the expected consequences of tES intervention, an appropriate stimulation strategy can be suggested. Different hypothetical examples for three neurochemical levels are presented below. For each example, some neuroscience and brain stimulation evidence are given first and then a protocol is suggested accordingly.

**• Neurochemical/Local**

- Neuroscience Evidence:
  ▪ “GABA level is abnormally increased in region A in patients with disorder X”.

- Stimulation Evidence:
  ▪ “Excitatory (anodal) tDCS (1 mA for 10 min, left M1/contralateral supraorbital ridge montage) causes locally reduced GABA neuronal activity (Stagg et al., 2009).

- Suggested Protocol: *Anodal tDCS over region A*.

**• Neurochemical/Small-scale Networks**

- Neuroscience Evidence:
  ▪ “Dopaminergic activity in the striatum, modulated by midbrain neurons, is dysfunctional in disorder X”.

- Stimulation Evidence:
  ▪ “Anatomical studies on monkeys show projections of the PFC to the caudate nucleus and striatum (Kemp and Powell, 1970; Selemon and Goldman-Rakic, 1985). PET imaging revealed release of dopamine in the head of the striatum evoked by excitatory (high frequency) rTMS application over the left mid-dorsolateral PFC (Strafella et al., 2001). Also, tDCS of the PFC (2 mA for 15 min, anode over ventromedial PFC, cathode over dorsolateral PFC) activates remote midbrain centers (Chib et al., 2013).”

- Suggested Protocol: *Anodal tDCS over the PFC to induce dopamine release in the striatum through cortico-subcortical pathways*.

**• Neurochemical/Large-scale Networks**

- Neuroscience Evidence:
  ▪ “Glutamate and GABA neurotransmitters have a basic role in neuroplasticity and their concentration mediates activation and deactivation of large-scale networks in the brain (Vidal-Piñeiro et al., 2015). Dysfunction of neuroplasticity and glutamate/GABA microcircuits within the default mode network are reported in the disorder X”.

- Stimulation Evidence:
  ▪ “MRS imaging has revealed the ability of excitatory Theta burst stimulation over the left inferior parietal lobule, one of the default mode network nodes, to modulate GABA within this network (Vidal-Piñeiro et al., 2015)”.

- Suggested Protocol: *Anodal tDCS over the left inferior parietal lobule in order to balance glutamate/GABA concentration in disorder X*.

#### Hypothetical tES Protocols Based on the Neuroelectrical Level of the Spatiomechanistic Framework

Some hypothetical examples for the neuroelectrical level of the proposed spatiomechanistic framework are provided in this section.

**• Neuroelectrical/Local**

- Neuroscience Evidence:
  ▪ “Activity of cortical region A is pathologically increased in disorder X”.

- Stimulation Evidence:
  ▪ “Cathodal tDCS (e.g., 1 mA for 4 s, motor cortex/supraorbital ridge montage, Nitsche and Paulus, 2000) can diminish cortical excitability, promote intracortical inhibition, and induce LTD-like effects (Nitsche and Paulus, 2000, 2001; Nitsche et al., 2003b, 2005)”.

- Suggested Protocol: *Application of cathodal tDCS to region A to reduce excitability of this hyperactive region*.

**• Neuroelectrical/Small-scale Networks**

- Neuroscience Evidence:
  ▪ “Balance of inhibitory connections of the right and left cortical regions, *A*_right_ and *A*_left_, via the corpus callosum is disturbed in disorder X, resulting in abnormal hypo-activity of *A*_right_ and hyper-activity of *A*_left_”.

- Stimulation Evidence:
  ▪ “Cathodal tDCS can diminish cortical excitability, promote intracortical inhibition, and induce LTD-like effects. Anodal stimulation, on the other hand, causes neuronal depolarization, and can lead to an increase of excitability (e.g., 1 mA for 4 s, motor cortex/contralateral supraorbital ridge montage; Nitsche and Paulus, 2000, 2001; Nitsche et al., 2003b, 2005)”.

- Suggested Protocol*: Bilateral stimulation of the A regions (left cathodal/right anodal, a bilateral bipolar-balanced montage) to counteract this pathological dysbalance*.

**• Neuroelectrical/Large-scale Networks**

**- Neuroscience Evidence:**
  ▪ “Resting-state fMRI and modularity network analysis show impaired interactions between the salience network, default-mode network, and executive control network in disorder X. The salience network pathologically allocates attentional resources towards internal stimuli, which leads to abnormally enhanced activity of the default-mode network and decreased activity of the executive control network”.

**- Stimulation Evidence:**

▪ “tDCS-fMRI studies have revealed the ability of tDCS to reconfigurate large-scale brain network activity; specifically bilateral tDCS over dorsolateral PFC regions (2 mA for 20 min, anode over the right dorsolateral PFC and cathode over the left dorsolateral PFC and vice versa) decreased activity of the default-mode network (Peña-Gómez et al., 2012; Monfared et al., 2014) and increased activity of the anticorrelated network (executive control network; Peña-Gómez et al., 2012)”.

- Suggested Protocol: *Bilateral stimulation over the dorsolateral PFC (a bilateral bipolar-balanced montage) to scale down the activity of the default-mode network and increase the activity of the executive control network in the disorder X*.

#### Hypothetical tES Protocols Based on the Oscillatory Level of the Spatiomechanistic Framework

Some hypothetical examples for the neuroelectrical level of the proposed spatiomechanistic framework are provided in this section.

**• Oscillatory/Local**

▪ ***Neuroscience Evidence:*** On one hand, deficient response inhibition is considered to be the primary deficit and the major characteristic of disorder X; on the other hand evidence suggests an association between behavioral inhibition and theta band activity in the right inferior frontal gyrus. Furthermore, some of the available pharmacological treatments for disorder X have been shown to decrease the absolute and relative power of theta band in the right inferior frontal gyrus.
▪ ***Stimulation Evidence:*** In a population of healthy participants, anodal tDCS over the right inferior frontal gyrus coupled with cathodal tDCS over the left orbitofrontal cortex (1.5 mA for 15 min) induced a selective reduction in the power of theta band in the right inferior frontal gyrus area (Jacobson et al., 2012) associated with improved behavioral inhibition (Jacobson et al., 2011).
▪ ***Suggested Protocol:*** *Application of the same protocol (which is a bilateral bipolar-nonbalanced one) might be beneficial for regulating theta band activity and improving behavioral inhibition deficits in patients with disorder X*.

**• Oscillatory/Small-scale Networks**

▪ ***Neuroscience Evidence:*** Evidence shows pathological beta oscillations in the deep region A in patients with disorder X associated with specific clinical symptoms.
▪ ***Stimulation Evidence:*** Both pharmacological and deep brain stimulation treatment for disorder X diminish beta-band activity in region A. This suppression is associated with improvement of related clinical symptoms. Beta activity of region A has been shown to be negatively correlated with alpha activity in cortical region B. Anodal tDCS has been able to enhance alpha activity in region B. Moreover, excitatory rTMS over cortical region B has been shown to reduce beta-band activity in region A in these patients.
▪ ***Suggested Protocol:*** *Application of anodal tDCS over the cortical region B to modulate beta oscillations in the deep region A*.

**• Oscillatory/Large-scale Networks**

▪ ***Neuroscience Evidence:*** A lack of resting state low frequency alpha activation in the default-mode network has been shown in a group of awake, relaxed patients with disorder X. This aberrant default-mode network activity has been associated with cognitive impairments in these patients.
▪ ***Stimulation Evidence:*** Low frequency rTMS over one of the main nodes of the default-mode network (right or left angular gyrus) increases resting-state alpha power density in the neural regions involved in this network (Capotosto et al., 2014).
▪ ***Suggested Protocol:*** *Cathodal tDCS over the same nodes (right or left angular gyrus) of the default-mode network with an extracephalic return electrode (a bilateral multiple monopolar montage) to increase the power of respective alpha rhythm*.

### Tailoring Based on the Various Subtypes (Clusters) of a Brain Disorder

The next stage of the protocol definition/selection based on the proposed framework is to define protocols based on clusters/subtypes of a respective brain disorder (*Tailoring based on the Various Subtypes (Clusters) of a Brain Disorder*). Obtained information from efforts for delineating subtypes of different disorders such as auditory hallucinations (McCarthy-Jones et al., 2014), and tinnitus (Landgrebe et al., 2010) based on their neurobiological characteristics, etiology, pathophysiology, and symptoms can provide valuable insights to select between different treatment options and individualize treatment approaches in the future.

As an example of adjusting the treatment target based on a disorder subtype, we refer to Parkinson’s disease. Parkinson’s disease has been clustered to tremor dominant and non-tremor dominant akinetic-rigid subtypes based on the predominant motor sign. FMRI, post-mortem analyses and voxel-based morphometry have revealed functional and structural differences in the patients with tremor dominant vs. non-tremor dominant akinetic-rigid phenotypes. Specifically, non-tremor dominant akinetic-rigid patients show a reduced BOLD signal compared to tremor dominant patients in the thalamus and specific nuclei of the basal ganglia (internal globus pallidus, external globus pallidus; Prodoehl et al., 2013). Although the optimal target point for deep brain stimulation in Parkinson’s disease is still a matter of debate, this clustering has been useful in the selection of anatomical targets. As tremor cells are located the lateral portion of the ventral intermediate nucleus (Brodkey et al., 2004; Katayama et al., 2005), in patients with tremor-dominant Parkinson’s disease, thalamic deep brain stimulation of the ventral intermediate nucleus can effectively alleviate parkinsonian tremor (Benabid et al., 1996; Schuurman et al., 2008). Complete and immediate suppression of tremor is usually achieved using continuous stimulation of the ventral intermediate nucleus at a high frequency (Benabid et al., 1996). In contrast to being highly beneficial for tremor control, ventral intermediate thalamic deep brain stimulation is ineffective for the other disabling features of Parkinson’s disease including bradykinesia, rigidity and gait and postural disturbances. Subthalamic nucleus and globus pallidus internus have been selected as alternative targets in deep brain stimulation treatment of Parkinson’s disease. Subthalamic nucleus and internal globus pallidus stimulation are very effective in dyskinesia reduction and also improving other symptoms (Limousin-Dowsey et al., 1999). Similar categorizations might be applicable when employing tES interventions. To the best of our knowledge, there is no study which has used two different tES protocols for different clusters within a brain disorder. This might, however, be an important issue for further investigations.

### Tailoring Based on Individual-Level Data

One step forward, which might be considered as a part of future progression towards precision tES, is to collect data from each individual patient using different techniques to characterize individual variability. This way, a higher level of individualization might be achieved and used to focus on treating individual patients rather than treating a certain disease (*Tailoring based on Individual-level Data*). Considerable inter- and intra-individual variability in response to tES currently limits tES translation from research to clinical practice. Multiple mechanisms contribute to this inter-individual variability, including genetics, gender, age, head anatomy, hormone levels and time of day of intervention. Just focusing on stimulation dosage, studies have reported dose-dependent significant differences of tES after-effects. In 2015, for instance, Chew et al. (2015) investigated the effects of anodal tDCS (anode/cathode positioned over the left primary motor cortex/right supraorbital area) using four different current intensities (0.2, 0.5, 1 and 2 mA) during five sessions (two sessions with 0.5 mA current amplitude). By investigating changes of motor evoked potential (MEP) amplitudes, they observed significant inter- and intra-individual variability in response to tDCS; e.g., in 28% of subjects, none of the current intensities induced an excitatory response. 67%, 19% and, 14% of the remaining subjects had an excitatory response to only one, two and all of the current intensities applied, respectively. Significant intra-individual variability in responses was also found; i.e., the outcomes of two identical 0.5 mA sessions were not similar at an individual level. Their results also showed a non-linearity of tDCS effects as a function of current intensity, as 0.5 mA stimulation intensity was less effective in inducing an excitatory or inhibitory response compared to both 0.2 mA and 2 mA (Chew et al., 2015). However, intra-individual variability has been suggested to be lower than inter-individual variability. By controlling for some variability-inducing parameters such as attention level, anxiety and time of the day, Jamil et al. (2017) observed good reproducibility in the cortical excitability modulation by anodal tDCS over three sessions (1 mA, 15 min, motor cortex/contralateral supraorbital electrode montage). Reliability of intra-individual responses to tDCS has also been shown in other studies (López-Alonso et al., 2015).

Moliadze et al. (2015) showed a dose- and age-dependency of tDCS effects by applying 1 mA and 0.5 mA anodal, cathodal, or sham tDCS (10 min) over the motor cortex of pediatric participants and measuring MEP amplitudes. Both the direction and durability of tDCS-induced after-effects were different in children as compared to adults. The direction of anodal after-effects (increase in MEP amplitudes) corresponded well with those observed in adults; however, MEP amplitudes not returning back to the baseline 1 h after tDCS suggests longer-lasting after-effects in children. On the other hand, 1 mA cathodal stimulation, in contrast to the results of the majority of previous studies conducted in adults, increased cortico-spinal excitability in children (Moliadze et al., 2015). In line with this study, results of simulations suggest larger electrical fields at the cortical surface in children than in adults induced by identical stimulation protocols (Kessler et al., 2013).

Various kinds of data might be useful to tract heterogeneity-inducing sources of tES effects which then can be leveraged to individualize therapy. As suggested by the proposed spatiomechanistic framework, different techniques might be employed for individualized data acquisition (Figure 3). Focusing on brain mapping techniques, data using TMS, fMRI, MEG, and EEG can provide information to decide about “neuroelectrical” alterations of the brain of a specific patient. MRS and PET data can be relevant when assessing “neurochemical” abnormalities in different spatial levels of a specific patient’s brain. MEG, EEG and specifically topographic quantitative EEG provide the opportunity to record brain oscillations for a specific patient which then can be analyzed in a particular region, be employed to assess synchronization/correlation/coherence between two regions, or evaluate rhythmic patterns across the whole brain. After profiling and gathering relevant data as brainprints of a specific patient’s disorder, these can be used to establish the foundation of a protocol design tailored for the individual patient.

The personalization step in tES protocol tailoring is probably more or less a story for the future, because our knowledge about the factors that determine individual efficacy is limited at present. There are, however, some studies which might be considered in this regard. For instance, in a tDCS study targeting patients with refractory epilepsy, knowledge about the disorder and action mechanisms of brain stimulation might narrow down the protocol search space to the local/neuroelectrical level of our framework. Then, utilizing EEG to record personal neuroelectrical data, one might progress toward the next stages of individualization and define a participant-specific stimulation protocol. Specifically, the cortical-excitability-diminishing cathode can be applied to the epileptogenic focus defined according to the individual EEG and the anode positioned over an area without epileptogenic activity (or a so-called silent area; Fregni et al., 2006c). Furthermore, individual responsiveness to TMS pulses might provide a useful measure for adjusting tES intensity (Labruna et al., 2016).

New structural connectivity techniques such as diffusion tensor imaging and various network and connectivity analysis methods in combination with the previously mentioned data can give rise to a large volume of information about local areas, small networks, and whole brain levels, and generate some useful data for all three classes (neuroelectrical, neurochemical, and oscillatory). Furthermore, physiologically validated modeling approaches can provide pivotal complementary help in tailoring stimulation parameters to overcome interindividual variability. We pointed out two categories of models employed in tES studies: (1) biophysical models of the head and electrodes; and (2) network/neuronal models.

Biophysical models can be used to individually calculate/predict current density distributions and adapt the stimulation parameters according to the structural and functional features of individual subjects. These models can help to: (1) tailor electrode montage and stimulation protocols to affect the brain regions of interest; (2) normalize stimulation parameters based on individual variations (both for healthy subjects and patients); (3) customize stimulation parameters for potentially vulnerable populations (e.g., people with skull damage, children); (4) design novel electrode shapes and electrode montages (e.g., for improved spatial focality); (5) assess and quantify current distribution and densities when using novel electrode shapes and/or montages; (6) consider compliance with safety guidelines; and (7) interpret patient-specific results. These computational models have the potential to act as a starting point to help in designing safe and effective electrotherapies, specifically for disorders in which brain structure changes are relevant (e.g., stroke or addiction, da Silva et al., 2013); but before that they require direct experimental and physiological validation (Datta et al., 2013). Also, it should be noted that because of the highly non-linear nature of neural functioning, modeled physical effects might not translate one-to-one into physiological and functional effects.

At another level, network models can be useful to anticipate neural effects of different stimulations with various amplitudes and frequencies (Reato et al., 2010). The suggested superior effectiveness of individualized protocols based on computational models has however to be validated in the basic and clinical experimental studies, including well-designed clinical trials.

## Summary

In this article, we propose a neuroscience-informed spatiomechanistic framework which can be used for tES protocol design. It can be beneficial for selection of a potentially efficient protocol for a specific brain disorder. Furthermore, it can be employed for exploration of various, even untouched, tES protocols, and individualized protocol design. We described how the proposed framework can provide a rationale to build hypotheses about physiologically-based optimized/tailored stimulation protocols in three stages: (1) tailoring based on the group-level data of a brain disorder; (2) various clusters of patients with a brain disorder; and (3) individual-level data. Various challenges must be addressed to bring the ambitious goal of providing precision tES therapy for patients in routine clinical settings to fruition. To begin with, high-quality characterizing information must be obtained consistently in the diagnostic setting. Also, dependent on the availability of relevant knowledge bases, it is crucial to take action based on the obtained data. Different questions persist regarding the extent of required data, the cost-effectiveness of various paradigms, and how rapidly various data can be delivered for treatment individualization. Further longitudinal studies are necessary to determine whether and how these neurophysiological and neuroimaging data might act as biomarkers for fingerprinting of the respective brain disorders.

Beyond treatment selection, the proposed framework can also help to generate ideas about treatment effect monitoring. Regular monitoring of response to therapy is crucial to be sure about the efficiency of treatment, absence of adverse effects and to facilitate treatment completion. Depending on the selected level of the respective framework, it can help to define appropriate human brain mapping methods and parameters of interest for assessment of the response and to decide how and where to monitor tES treatment effects in a mechanistic way and address different responses to determine treatment fidelity.

Closed loop tES, i.e., online adjustment of stimulation parameters according to “intra subject” dynamic brain states, might be the final step on this road and is currently under development. One example is to adaptively change tACS frequency based on frequency information derived from EEG recordings (Boyle and Frohlich, 2013; Wilde et al., 2015). Future investigations focusing on other neuroimaging and electrophysiological data and other control parameters such as electrode positions are required. Although online adjustment of electrode positions appears difficult to implement, employing several electrodes and activating the proper ones in a feedback-based manner might provide a solution.

## Author Contributions

FY, MAN and HE made substantial contributions to the conception of the work, as well as drafting and revising it critically for important intellectual content and final approval of the version to be published; agree to be accountable for all aspects of the work in ensuring that questions related to the accuracy or integrity of any part of the work are appropriately investigated and resolved; came up with the idea and wrote the article.

## Conflict of Interest Statement

MAN is in the Advisory Board of Neuroelectrics. The other authors declare that the research was conducted in the absence of any commercial or financial relationships that could be construed as a potential conflict of interest.

## Abbreviations

BDNF: Brain-derived Neurotrophic Factor
BOLD: Blood-oxygen-level Dependent
f/MRI: functional/Magnetic Resonance Imaging
GABA: gamma-Aminobutyric acid
LTP: Long-term Potentiation
LTD: Long-term Depression
MEG: Magnetoencephalography
MEP: Motor Evoked Potentials
PET: Positron Emission Tomography
PFC: Prefrontal Cortex
rTMS: repetitive Transcranial Magnetic Stimulation
tACS: transcranial Alternating Current Stimulation
tDCS: transcranial Direct Current Stimulation
tES: transcranial Electrical Stimulation
tRNS: transcranial Random Noise Stimulation.
